# Integration of genome wide association studies and whole genome sequencing provides novel insights into fat deposition in chicken

**DOI:** 10.1038/s41598-018-34364-0

**Published:** 2018-11-01

**Authors:** Gabriel Costa Monteiro Moreira, Clarissa Boschiero, Aline Silva Mello Cesar, James M. Reecy, Thaís Fernanda Godoy, Fábio Pértille, Mônica Corrêa Ledur, Ana Silvia Alves Meira Tavares Moura, Dorian J. Garrick, Luiz Lehmann Coutinho

**Affiliations:** 1 Department of Animal Science, University of São Paulo, Piracicaba, SP Brazil; 2 Department of Animal Science, Iowa State University, Ames, IA USA; 3 Embrapa Suínos e Aves, Concórdia, SC Brazil; 4 FMVZ/São Paulo State University - UNESP, Botucatu, SP Brazil; 5 School of Agriculture, Massey University, Ruakura, Hamilton, New Zealand

## Abstract

Excessive fat deposition is a negative factor for poultry production because it reduces feed efficiency, increases the cost of meat production and is a health concern for consumers. We genotyped 497 birds from a Brazilian F_2_ Chicken Resource Population, using a high-density SNP array (600 K), to estimate the genomic heritability of fat deposition related traits and to identify genomic regions and positional candidate genes (PCGs) associated with these traits. Selection signature regions, haplotype blocks and SNP data from a previous whole genome sequencing study in the founders of this chicken F2 population were used to refine the list of PCGs and to identify potential causative SNPs. We obtained high genomic heritabilities (0.43–0.56) and identified 22 unique QTLs for abdominal fat and carcass fat content traits. These QTLs harbored 26 PCGs involved in biological processes such as fat cell differentiation, insulin and triglyceride levels, and lipid biosynthetic process. Three of these 26 PCGs were located within haplotype blocks there were associated with fat traits, five overlapped with selection signature regions, and 12 contained predicted deleterious variants. The identified QTLs, PCGs and potentially causative SNPs provide new insights into the genetic control of fat deposition and can lead to improved accuracy of selection to reduce excessive fat deposition in chickens.

## Introduction

In the past, slow growth rates were a challenge in poultry production systems; consequently, intensive selection of this trait in elite great-grand parent lines has dramatically increased poultry productivity. Modern commercial broiler chickens are produced from crosses that have been simultaneously selected for rapid growth, increased meat-production, and improved carcass yield^1,2^. However, chickens selected for higher body weight might exhibit increased appetite and excessive energy consumption, which may lead to excessive fat accumulation^3–5^.

Excessive fat deposition in chickens is a negative factor for meat production because it reduces feed efficiency and the value of the carcass^6^. Therefore, understanding the genetic architecture, uncovering genomic regions, and finding positional candidate genes (PCGs) associated with fat deposition related traits could be helpful in breeding programs.

A total of 200 quantitative trait loci (QTL) have been reported for abdominal fat weight, 139 for abdominal fat percentage and 11 for carcass fat content^7^. Previous QTL studies, performed in the same population evaluated in this study (Embrapa F_2_ Chicken Resource Population) using 128 microsatellite markers for 22 autosomal chromosomes, mapped QTLs for abdominal fat traits^8–10^ and carcass fat traits^9^, but the genomic locations of these QTL had large confidence intervals. Genotyping of animals using high-density marker arrays can help the identification of genomic regions with smaller intervals^11^, which, in turn, facilitates the identification of candidate genes.

Many studies have been performed to identify candidate genes for fat deposition traits in chicken^12–15^, but the discovery of a causal mutation is still a challenge. In a study with chicken, candidate genes for egg production and feed efficiency were identified by genome-wide association study (GWAS), and this information was integrated with selection signature results highlighting important biological processes and causal variants^16^. However, to the best of our knowledge, no integrative studies were performed to identify potential causal genes and mutations for fat deposition regulation.

Thus, the aims of this study were to estimate genomic heritability for different fat deposition related traits and identify genomic regions and PCGs in an F_2_ population from a cross between a broiler line and an egg-layer line. We also integrated these results to refine our list of candidate genes using selection signature regions and SNP data information previously obtained from whole genome sequence of the parental generation evaluated in this study.

## Results

### Genotyping and quality control

From the 529 genotyped chickens, 40 were removed before GWAS analysis. Of these, 12 were removed because their genotypes did not pass quality control (DishQC ≥0.82 and call rate ≥90% filter), and 28 did not have complete phenotypic data. As a result, 489 F_2_ chickens were used for the association analysis.

From the 580,961 SNPs originally available on the chicken SNP array, 399,693 SNPs segregating in the F_2_ population were kept for further analysis. A total of 4,304 were removed due to low minor allele frequency (MAF ≤0.02), and 23,603 SNPs located in sex chromosomes and unmapped linkage groups were also removed. After these filtering criteria, 371,786 SNPs from the autosomal chromosomes (GGA1-28) remained for the GWAS analysis. The average genotype density per chromosome was 541 SNPs/Mbp, with the lowest density being observed on GGA2 (297 SNPs/Mbp), and the highest density on GGA21 (816 SNPs/Mbp).

### Descriptive Statistics

The number of animals, mean and standard deviations, variance components and estimated genomic heritabilities are presented in Table 1. Genomic heritability values ranged from 0.43 for carcass fat content (CFC) to 0.56 for abdominal fat percentage (ABFP).

**Table 1:** Descriptive statistics, variance components and genomic heritability.

Trait	N	Average ± SD^a^	Genetic variance	Residual variance	Total variance	Genomic heritability^b^
ABF	476	15.60 ± 7.26	11.481	10.301	21.782	0.47
ABFP	476	1.56 ± 0.60	0.133	0.169	0.302	0.56
CFC	451	145.35 ± 40.52	212.237	159.809	372.047	0.43
CFCDM	451	39.75 ± 4.63	8.404	9.859	18.263	0.54

ABF: abdominal fat weight in grams; ABFP: abdominal fat percentage; CFC: carcass fat content in grams; CFCDM: carcass fat content on dry matter basis.

^a^Means and standard errors.

^b^Genomic heritability estimated with a Bayes B model.

### Genome-wide association analysis (GWAS)

The genomic windows associated with fat traits are detailed in Table 2. Twenty-two significant unique 1 Mb windows (based on genome position) present on GGA1, 2, 7, 15, 20, 27 and 28 were identified. The posterior probability of association (PPA), as described by Onteru *et al*.^17^, ranged from 0.41 to 0.85 for these regions, and the percentage of genetic variance explained by the windows ranged from 0.53 to 1.71.

**Table 2:** Characterization of 1 Mb genomic windows associated with abdominal fat and carcass fat content traits in the Embrapa F_2_ Chicken Resource Population.

Trait	GGA_Mb^a^	Genome interval (start – end position)^a^	N° of SNP/window	% genetic variance explained	PPA^b^	SNP ID^c^	Model frequency
ABF	1_52	52,000,127–52,998,004	387	1.23	0.56	rs313050579	0.0108
	1_53	53,002,697–53,997,943	282	1.32	0.56	rs312317108	0.0158
	1_54	54,001,671–54,998,619	257	0.75	0.42	rs15271198	0.0173
	1_179	179,001,074–179,999,169	411	0.67	0.54	rs13557213	0.0111
	2_30	30,004,050–30,999,519	315	0.63	0.47	rs317553502	0.0138
	2_61	61,003,805–61,992,322	290	0.94	0.52	rs13619262	0.0527
	2_62	62,001,908–62,998,786	307	1.23	0.56	rs314667858	0.0253
	27_3	3,000,222–3,996,811	820	0.84	0.77	rs315719114	0.0097
	28_4	4,004,758–4,964,406	629	0.53	0.68	rs314073448	0.0038
ABFP	2_61	61,003,805–61,992,322	290	0.71	0.57	rs13619262	0.0272
	2_62	62,001,908–62,998,786	307	0.66	0.47	rs14193698	0.0193
	7_35	35,001,761–35,996,723	386	0.58	0.58	rs312894632	0.0106
	28_0	23,942–999,295	829	1.09	0.80	rs316394502	0.0512
	28_3	3,000,142–3,988,940	621	0.82	0.73	rs15251024	0.0146
CFC	1_53	53,002,697–53,997,943	282	0.91	0.51	rs314857319	0.0083
	1_168	168,005,668–168,997,872	318	0.56	0.41	rs312378109	0.0093
	1_169	169,001,420–169,999,438	346	0.82	0.44	rs315077363	0.0109
	1_170	170,002,808–170,999,129	446	0.98	0.48	rs13973557	0.0223
	1_171	171,000,120–171,999,874	407	0.91	0.52	rs315852521	0.0142
	1_175	175,003,078–175,996,880	405	1.28	0.52	rs313574684	0.0246
	7_35	35,001,761–35,996,723	386	0.73	0.52	rs314947533	0.0125
	7_36	36,000,235–36,898,384	257	0.58	0.44	rs312848275	0.0158
	15_9	9,002,743–9,999,015	639	0.82	0.64	rs316091564	0.0637
	15_10	10,001,717–10,999,147	577	0.71	0.63	rs13528818	0.0156
	28_4	4,004,758–4,964,406	629	0.82	0.75	rs314212680	0.0069
CFCDM	1_105	105,000,541–105,997,476	383	1.16	0.60	rs13916775	0.0154
	1_175	175,003,078–175,996,880	405	1.49	0.60	rs313574684	0.0206
	1_179	179,001,074–179,999,169	411	0.89	0.53	rs317863254	0.0121
	7_35	35,001,761–35,996,723	386	0.95	0.58	rs16614131	0.0170
	7_36	36,000,235–36,898,384	257	1.23	0.57	rs312848275	0.0257
	20_12	12,000,087–12,998,691	562	0.54	0.67	rs739732531	0.0204
	28_3	3,000,142–3,988,940	621	0.76	0.62	rs315921612	0.0176
	28_4	4,004,758–4,964,406	629	1.71	0.85	rs313086976	0.0181

ABF: abdominal fat weight in grams; ABFP: abdominal fat percentage; CFC: carcass fat content in grams; CFCDM: carcass fat content on dry matter basis.

^a^Map position based on Gallus_gallus-5.0, NCBI assembly.

^b^Posterior probability of association (PPA) as described by Onteru et al.^17^.

^c^SNP within the window with the highest model frequency.

Many SNPs were fitted simultaneously in the model (see Methods), and due to high linkage disequilibrium, the QTL effect will be distributed across these markers, shrinking the SNPs effects at individual loci^18^. In order to identify the SNPs within the windows most probably linked to the QTL, we selected those with highest model frequency (Table 2), as adopted by Van Goor *et al*.^19,20^. The characterization of those SNPs including their effect, frequency in the population and frequency in the founder lines (TT and CC) is available in Supplementary Spreadsheet S1.

**Figure 1:**
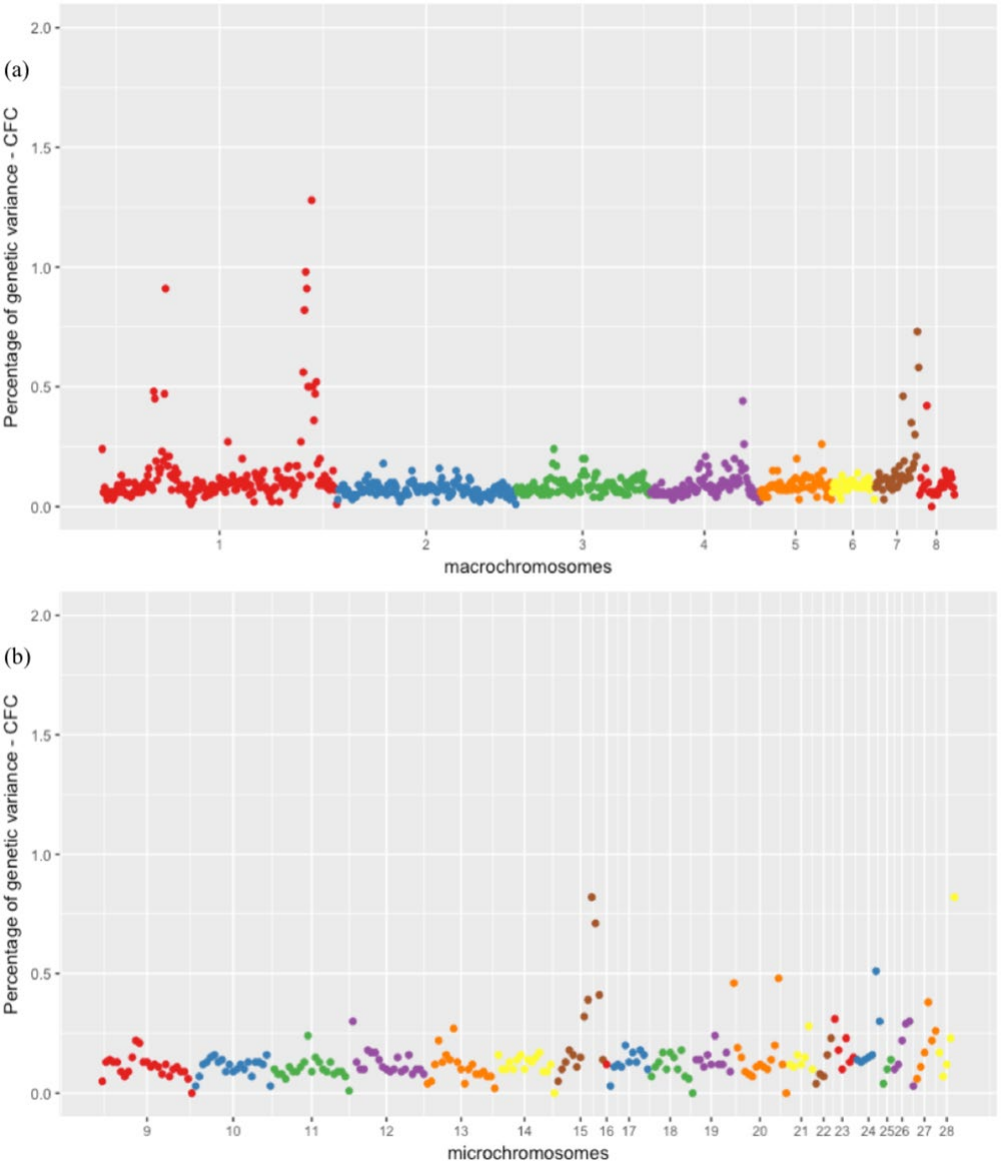
Manhattan plot of the posterior means of the percentage of genetic variance explained by each 1 Mb SNP window across the 28 autosomal chromosomes for CFC (carcass fat content in grams): (**A**) genomic windows located on macrochromosomes, and (**B**) genomic windows located on microchromosomes.

Carcass fat content exhibited the highest number of significant QTLs (Fig. 1), followed by ABF, CFCDM and ABFP (Supplementary Figs S1, S2 and S3). For CFC, the region that explained the largest amount of genetic variation was on GGA1 (175 Mb), with 1.28% of the genetic variance. For ABF, the region was on GGA1 (53 Mb), with 1.32% of the genetic variance. For CFCDM, it was on GGA28 (4 Mb), with 1.71% of the genetic variance, and for ABFP, it was on GGA28 (0 Mb), with 1.09% of the genetic variance.

Adjacent significant windows were considered as likely representing the same QTL, and their respective percentages of genetic variance explained were summed. For abdominal fat traits, three adjacent windows on GGA1 (52–54 Mb) were associated and cumulatively accounted for 3.3% of the genetic variance for ABF, and two adjacent windows on GGA2 (61–62 Mb) cumulatively accounted for 2.17% and 1.37% of the genetic variance for ABF and ABFP, respectively (Table 2).

For carcass fat content traits, four adjacent windows on GGA1 (168–171 Mb) were associated and cumulatively accounted for 3.27% of the genetic variance for CFC; two adjacent windows on GGA7 (35–36 Mb) cumulatively accounted for 2.18% and 1.31% of the genetic variance for CFC and CFCDM, respectively; two adjacent windows on GGA15 (9-10 Mb) cumulatively accounted for 1.53% of the genetic variance for CFC; and two adjacent windows on GGA28 (3-4 Mb) cumulatively accounted for 2.47% of the genetic variance for CFCDM (Table 2).

Co-located QTLs (the same QTL associated with abdominal fat and carcass fat content traits) were identified on GGA1 at 53 Mb, GGA1 at 179 Mb, GGA7 at 35 Mb and on GGA28 (3-4 Mb).

### Overlap with previously reported QTLs

The 22 QTL regions identified in our study overlapped with 56 previously published QTLs for fatness traits mapped in different populations. The QTL on GGA28 at 0 Mb is novel (Table 3).

**Table 3:** Overlap of QTLs identified in our study with previously fatness QTLs reported from the Chicken QTLdb^7^.

GGA (Mb)	Known QTLs for fatness traits^a^
1 (52)	**ABF** (#6806, #12478); **ABFP** (#3350); **SCFT** (#14359); **CFWID** (#14360); **VISAT** (#17319); **SCNF** (#17325); **TWF** (#17332)
1 (53)	**ABF** (#6806, #6869, #9665, #12478); **ABFP** (#3350, #12590); **SCFT** (#14359); **CFWID** (#14360); **VISAT** (#17319); **SCNF** (#17325); **TWF** (#17332); **CFCDM** (#17117)
1 (54)	**SCFT** (#14359); **CFWID** (#14360); **VISAT** (#17319); **SCNF** (#17325); **TWF** (#17332); **CFCDM** (#17117)
1 (105)	**ABF** (#6858, #12466, #14361); **ABFP** (#14362); **SCFT** (#14359); **VISAT** (#17319); **SCNF** (#17325); **TWF** (#17332); **CFC** (#17119)
1 (168-172)^b^	**ABF** (#7010, #66054); **ABFP** (#7011); **TWF** (#17332); **CFC** (#17129); **CFCDM** (#17120, #17130); **SFWT** (#1856)
1 (175)^b^	**ABF** (#7010); **CFC** (#17129); **CFCDM** (#17120, #17130); **SFWT** (#1856)
1 (179)^b^	**ABF** (#7010); **CFC** (#17129); **CFCDM** (#17120, #17130); **SFWT** (#1856)
2 (30)	**VISAT** (#17320); **SCNF** (#17326, #17327); **TWF** (#17333)
2 (61-63)	**VISAT** (#17320); **SCNF** (#17326, #17327); **TWF** (#17333)
7 (35)	**ABF** (#2167)
7 (36)	**ABF** (#2167)
15 (9-11)^b^	**ABF** (#2347, #9451, #12631); **ABFP** (#9450); **CFC** (#17122); **CFCDM** (#17121); **FATDIS** (#12645)
20 (12)	**ABF** (#19476, #30881); **ABFP** (#19477, #30882)
27 (3)^b^	**ABF** (#11809, #11817, #66072); **ABFP** (#3354, #11820, #11934); **CFC** (#17126, #17135); **CFCDM** (#17125); **IF** (#3360)
28 (0)	—
28 (3)	**ABF** (#2417, #12632); **SFWT** (#2418, #12641)
28 (4)	**ABF** (#2417, #12632); **SFWT** (#2418, #12641)

ABF: abdominal fat weight; ABFP: abdominal fat percentage; CFC: carcass fat content; CFCDM: carcass fat content on dry matter basis; CFWID: cingular fat width; FATDIS: fat distribution; SCFT: subcutaneous fat thickness (fat thickness under skin); SCNF: subcutaneous neck fat weight (subcutaneous neck adipose tissue); SFWT: skin fat weight; TWF: total white fat weight (total white adipose tissue); VISAT: visceral fat weight (visceral adipose tissue weight).

^a^Previously known QTLs were reported by QTL ID numbers available at Chicken QTLdb^7^ – release 33.

^b^Indicates that this genomic window overlaps with known QTLs mapped in the same population^9,10^.

Five of the detected QTLs overlapped with known QTLs for ABF, CFC and CFCDM mapped in the same F_2_ population^9,10^ used herein (Table 3). These known QTLs covered 1.9 Mb (#11817 and #11809), 23.1 Mb (#17129), 2.7 Mb (#17122), and 23.1 Mb (#17130 and #17120).

### Positional candidate genes, overlap with selection signature regions, haplotype blocks and screening of sequencing SNPs

From the 22 QTLs identified, 14 contained PCGs selected based on Gene Ontology terms related to fat deposition from literature records. Among these, four PCGs overlapped with selection signature regions (Table 4; Supplementary Fig. S4.) identified in a previous study from our group^21^. In that previous study, our group reported a genome-wide characterization of regions under selection based on the Fst method applied to the sequencing variants of 14 broilers and 14 layers (founders of Embrapa F_2_ Chicken Resource Population)^21^. One PCG (*CRY1*) was located 1.5 kb from a selection signature region (Table 4, Supplementary Fig. S4).

**Table 4:** Genomic windows associated with fat deposition traits that harbor positional candidate genes.

GGA (Mb)	PCG^a^	Ensembl gene ID^b^	Number of SNPs^c^	SNP density (SNPs/kb)^d^
1 (52)	*MB*	ENSGALG00000012541	221	60
1 (53)^e^	*CRY1* ^g^	ENSGALG00000012638	675	20
1 (54)^e^	*CHST11* ^f^	ENSGALG00000030607	2593	17
1 (168)	*HTR2A*	ENSGALG00000016992	535	21
	*RB1*	ENSGALG00000016997	1673	20
1 (170)	*FOXO1*	ENSGALG00000017034	835	13
1 (175)	*SLC7A1*	ENSGALG00000017085	559	30
2 (30)	*IL6*	ENSGALG00000010915	161	60
7 (36)^e^	*NR4A2* ^f^	ENSGALG00000012538	1961	27
	*GPD2* ^f^	ENSGALG00000012543	628	15
15 (9)	*PLA2G1B*	ENSGALG00000020989	145	88
	*SIRT4*	ENSGALG00000007244	314	71
15 (10)	*SELM*	ENSGALG00000025972	166	82
20 (12)	*DOK5*	ENSGALG00000007786	663	19
28 (0)	*SLC1A6*	ENSGALG00000000558	554	25
	*ANGPTL4*	ENSGALG00000000619	305	43
	*RAB11B*	ENSGALG00000000613	384	30
28 (3)	*STK11*	ENSGALG00000040008	738	22
	*GDF3*	ENSGALG00000003161	103	49
	*TM6SF2*	ENSGALG00000029015	214	65
	*SLC25A42*	ENSGALG00000002621	239	35
	*SLC5A5*	ENSGALG00000041932	244	54
	*SLC39A3*	ENSGALG00000020582	170	116
28 (4)^e^	*PIK3R2*	ENSGALG00000003428	275	15
	*INSR* ^f^	ENSGALG00000040758	578	17
	*SLC35E1*	ENSGALG00000003794	103	22

^a^Positional candidate genes.

^b^Ensembl gene ID based on Galgal5 (*Ensembl Genes 90 Database*).

^c^Number of SNPs annotated on the PCG.

^d^SNP density in the respective PCG.

^e^Indicates that this genomic window overlaps with a selection signature region^21^.

^f^Indicates that the positional candidate gene was annotated within a selection signature region.

^g^Indicates that this positional candidate gene was located 1.5 kb from a selection signature region.

**Figure 2:**
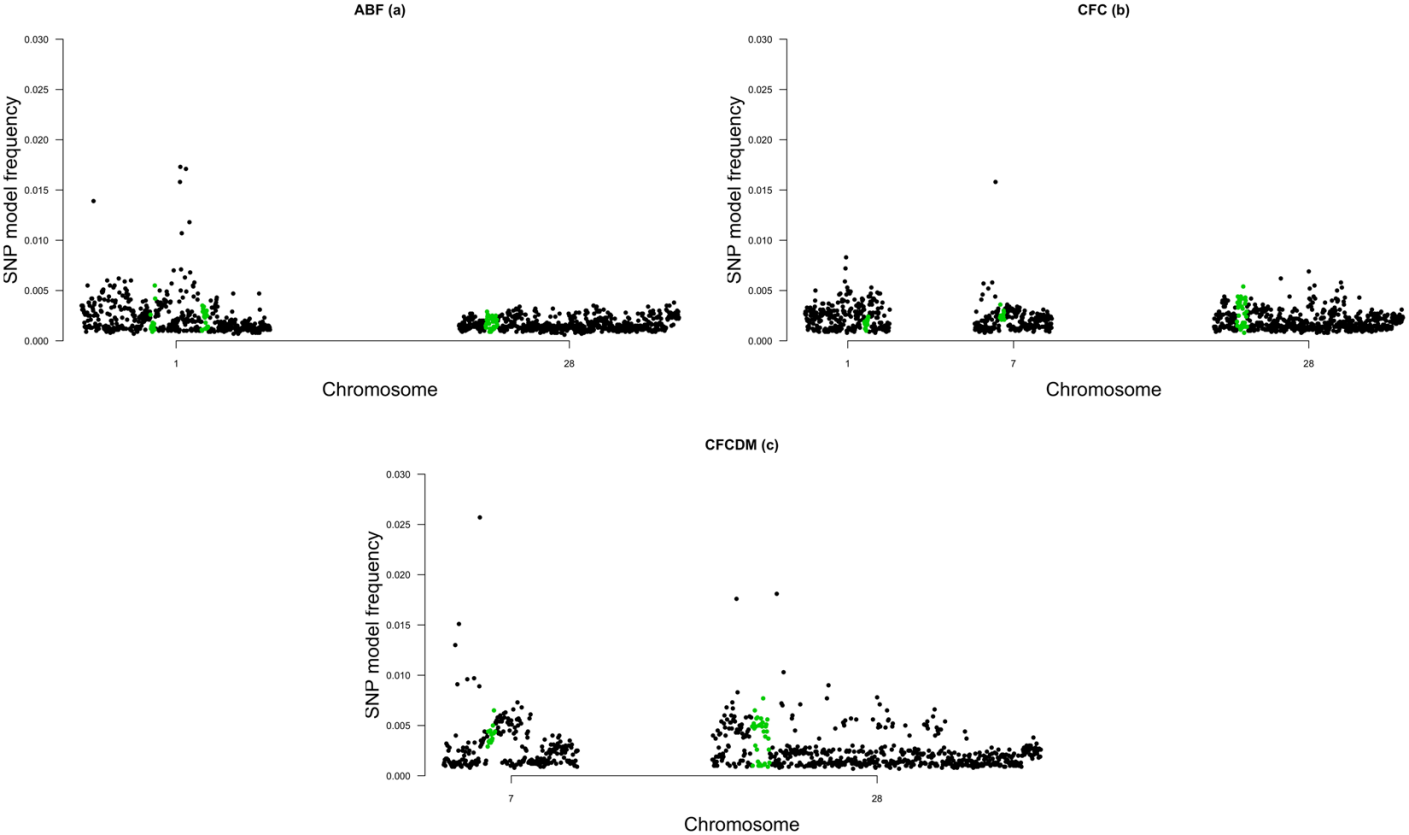
Manhattan plot of the SNP model frequencies distribution within each significant genomic window showing overlap with selection signature regions for: (**a**) abdominal fat weight (ABF); (**b**) carcass fat content (CFC); (**c**) carcass fat content on dry matter basis (CFCDM). The X-axis denotes the significant SNP window represented by the number of the respective chromosome and the Y-axis shows the model frequencies from Bayes B analysis.

In order to demonstrate further support for our findings, we checked the distribution of SNPs model frequencies within the genomic windows and their overlap with selection signature regions. Manhattan plots for ABF, CFC and CFCDM are presented in Fig. 2.

Those SNPs with the highest model frequency within the large effect genomic windows are likely to be in linkage disequilibrium with the candidate genes and causative mutation. Thus, the detection of haplotype blocks was performed and three PCGs were located within haplotypes blocks that harbored the SNPs with the highest model frequency within the associated genomic window. The characterization of the genomic windows, haplotype blocks and the overlapped PCGs are shown in Table 5. All of the haplotype blocks detected and the harboring SNPs with the highest model frequency in each associated genomic window can be found in Supplementary Spreadsheet S2.

**Table 5:** Characterization of the genomic windows and their respective haplotype blocks that encompass PCGs for fat deposition.

GGA_Mb^a^	Traits associated	SNP ID^b^	Haplotype blocks^c^		
			Start-End position^a^	Size (kb)	PCGs^d^
7_36	CFC, CFCDM	rs312848275	36,163,395–36,333,047	169.653	*NR4A2*, *GPD2*
28_4	CFCDM	rs313086976	4,111,155–4,174,053	62.899	*INSR*

CFC: carcass fat content in grams; CFCDM: carcass fat content on dry matter basis.

^a^Map position based on Gallus_gallus-5.0, NCBI assembly.

^b^SNP within the window with the highest model frequency.

^c^Haplotype block that harbor the SNP with the highest model frequency within the genomic window.

^d^Positional candidate genes located within the haplotype blocks.

**Figure 3:**
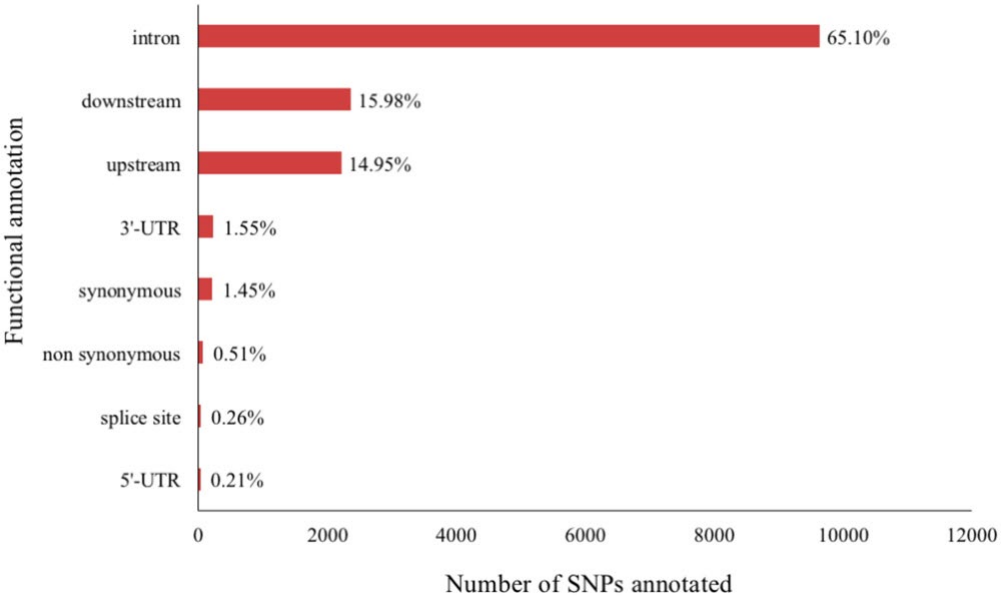
Functional annotation of unique SNPs identified in 26 PCGs. The percentage was estimated based on the total number of SNPs annotated in 26 PCGs.

Using the SNP data generated from the whole genome sequence of the founders of this population, we observed that 15,036 SNPs were located in the 26 PCGs. The annotation of these SNPs are in Fig. 3.

We also looked for potentially deleterious and high impact sequence SNPs, which may affect protein function or result in truncated proteins, based on SIFT score estimates and Ensembl prediction. Twelve potentially deleterious SNPs were identified in eight PCGs (Table 6), but none of the mutations in our PCGs were annotated as having high impact.

**Table 6:** Characterization of deleterious SNPs identified in eight PCGs.

Associated Gene Name	SNP ID	GGA	Genome position^a^	SIFT score^b^	Amino acid changed
*FOXO1*	g.170581941 > C/T	1	170,581,941	deleterious low confidence^c^ (0.01)	Pro/Leu
*NR4A2*	g.36224286 > C/T	7	36,224,286	deleterious (0)	Val/Met
	g.36225242 > G/T		36,225,242	deleterious (0)	Arg/Ser
	g.36225278 > C/T		36,225,278	deleterious (0.01)	Val/Met
*SIRT4*	rs316192467	15	9,435,175	deleterious (0.02)	Ala/Thr
*DOK5*	g.12473540 > C/A	20	12,473,540	deleterious (0.02)	Lys/Asn
*ANGPTL4*	g.846035 > G/A	28	846,035	deleterious (0.03)	Ser/Phe
*TM6SF2*	g.3553753 > T/C	28	3,553,753	deleterious low confidence^c^ (0.01)	Leu/Pro
	rs315426765		3,554,427	deleterious (0.05)	Leu/Phe
	rs741325985		3,554,836	deleterious (0.04)	Val/Met
*SLC39A3*	rs316529053	28	3,285,533	deleterious (0)	Arg/His
*SLC35E1*	g.4327417 > G/A	28	4,327,417	deleterious (0.05)	Gly/Arg

^a^Position based on *Gallus_gallus* 5.0 assembly.

^b^SIFT (Sorting Intolerant From Tolerant) score.

^c^Deleterious low confidence: little sequence diversity in this position affecting the substitution model and consequently, means of conservation value and the confidence of the prediction^74^.

## Discussion

### Genomic heritability

Genomic heritability was estimated using relationships inferred from high-density SNP panel genotypes instead of pedigree-based relationships. The use of close relatives and a higher density of SNPs may lead to a better genomic prediction with less bias^22,23^ than can be achieved using pedigree-based relationships.

For the evaluated traits, pedigree-based heritability estimates were found in the literature. In a study with 1,069 purebred full-sib male chickens, Chen *et al*.^24^ reported heritability estimates for ABF and ABFP of 0.62 and 0.24, respectively. Zerehdaran *et al*.^25^ reported heritability estimates for ABF and ABFP of 0.62 ± 0.09 and 0.71 ± 0.09, respectively, using 3,278 chickens from a meat-type population after nine generations of intercrossing. A higher value was reported for ABF (0.82) using the records of 300 chickens from a commercial female grandparent stock^26^, and could be explained by the fact that female broiler chickens generally deposit more abdominal fat than male broiler chickens^4,27^.

In the same population (Embrapa F_2_ Chicken Resource Population), pedigree-based heritability for ABF was estimated at 0.33 ± 0.19 in the F_2_-CTCT generation (layer males crossed with broiler females) and 0.82 ± 0.3 in the F_2_-TCTC generation (broiler males crossed with layer females)^28^. Considering the carcass fat content traits, heritability estimates for CFC expressed in percentage of wet carcass was 0.53 ± 0.10, and the heritability for fat percentage in dry-matter basis (CFCDM) was 0.55 ± 0.10, using records of 3,422 chickens^3^.

Genomic heritability estimates for ABF (0.33) and ABFP (0.31) were reported in one recent study performed with 1,408 chickens from a Brazilian broiler population under multiple trait selection^29^. Our genomic heritability estimates (Table 1) for fat deposition related traits were high (greater than 0.43), similar to the pedigree-based heritability reported for the same traits in the same F_2_ population. Therefore, a high proportion of the total variance for fat deposition traits is explained by genetic variance^22^, and selection against fat deposition can achieve good results in chicken.

### Genome-wide association (GWAS)

We observed that genetic variation explained by each 1 Mb genomic window ranged from 0.53 to 3.3% depending on the trait analyzed. Cumulatively, the significant windows associated with ABF, ABFP, CFC and CFCDM explained 8.14%, 3.28%, 9.12% and 8.73% of the genetic variance, respectively. Previous QTL mapping studies, using microsatellites markers in the same experimental population evaluated here, detected QTLs explaining 6.65%, 12.18%, 9.9% and 11% of the phenotypic variance for ABF, ABFP, CFC and CFCDM, respectively^9,10^, corroborating our findings. It is important to highlight that most of the genomic windows explained <0.53% of the genetic variance for the traits analyzed in our study (Supplementary Spreadsheet S3) and these percentages were not considered.

We performed the GWAS studies in a F2 experimental population generated from a cross between a white-egg type (layer, CC) line and a meat-type (broiler, TT) line^30^. These lines have a different background and previously underwent multi-trait selection for different traits for several generations, exhibiting considerable differences in growth and carcass traits^8,31^. According to Campos *et al*.^10^, chickens from this broiler line exhibit 15 fold more fat deposition than hens from the layer line reared as broilers.

Almost all the QTLs detected overlapped with previously reported QTLs associated with fat deposition traits in this or in other populations, confirming and refining previous results. The QTL located in the first bases of GGA28 was novel and might be a population specific QTL. Additionally, the QTL located on GGA27 at 3 Mb associated with ABF overlapped with a QTL previously mapped in the TT Reference population for chicken skin weight and percentage, used as indicators of subcutaneous fat^29^. The TT Reference population originated after 17 generations of multiple trait selection on birds from the broiler line (TT) used in the cross to obtain the Embrapa F_2_ Chicken Resource Population^32^. Thus, our findings indicate that even after many generations of multiple trait selection, the QTL located on GGA27 still has an effect on fat deposition whereas the other QTLs detected herein were fixed in the TT Reference population.

We compared the size of the QTL regions obtained in this study with a previous one that used 128 microsatellite markers in the same population^9,10^. For example, the QTL for ABF on GGA27 at 3 Mb overlapped with two known QTLs mapped to a region of 1.9 Mb (#11817 and #11809). The high-density SNP panel narrowed this region down to a 1 Mb interval, thus allowing a more focused search for candidate genes.

The QTLs mapped on GGA1 (at 53 and 179 Mb), GGA7 (35 Mb), and GGA28 (3-4 Mb) were associated with both abdominal fat and carcass fat content traits, indicating that they might be pleiotropic or reflect multiple causal mutations in the same QTLs.

We checked the overlap of our QTLs associated with fat deposition related traits and previously mapped QTLs for body weight at 35 (BW35) and also 41 (BW41) days-of-age mapped in the same F2 population^8,33–35^. From the 22 QTLs detected, 17 did not overlap with QTLs for body weight traits as expected since we included BW42 as a covariate in our analysis. The inclusion of BW42 as a covariate allowed the detection of 17 QTLs associated with fat deposition traits but with no phenotypic association with body weight traits. The five QTLs that overlapped with BW traits may have pleiotropic effects.

### Positional candidate genes for fat deposition

Positional candidate genes were selected based on their associated Gene Ontology (GO) terms and literature information. From the 26 PCGs selected, 17 showed GO terms for fat cell differentiation, insulin and triglycerides levels among other processes involved in fat deposition (Table 7).

**Table 7:** List of PCGs that exhibited GO terms related to lipid metabolic processes.

Gene	Gene Ontology terms
*CRY1*	response to insulin, lipid storage, glucose homeostasis
*HTR2A*	positive regulation of fat cell differentiation
*RB1*	regulation of lipid kinase activity
*FOXO1*	cellular response to insulin stimulus, insulin receptor signaling pathway, negative regulation of fat cell differentiation, glucose homeostasis
*IL6*	positive regulation of B cell activation
*NR4A2*	fat cell differentiation
*GPD2*	oxidation-reduction process, gluconeogenesis, glycerol-3-phosphate dehydrogenase activity
*PLA2G1B*	phospholipid metabolic process, lipid catabolic process, lipid metabolic process
*SIRT4*	negative regulation of insulin secretion, positive regulation of lipid biosynthetic process
*SELM*	adipose tissue development
*DOK5*	insulin receptor binding
*ANGPTL4*	triglyceride homeostasis, negative regulation of lipoprotein lipase activity
*RAB11B*	insulin secretion involved in cellular response to glucose stimulus
*STK11*	negative regulation of lipid biosynthetic process, glucose homeostasis
*TM6SF2*	regulation of lipid metabolic process
*PIK3R2*	cellular response to insulin stimulus, insulin receptor signaling pathway, cellular glucose homeostasis
*INSR*	cellular response to insulin stimulus, insulin binding, insulin-activated receptor activity, insulin-like growth factor receptor binding, insulin-like growth factor I binding, insulin-like growth factor II binding, insulin receptor substrate binding, insulin receptor signaling pathway, insulin receptor complex, glucose homeostasis, positive regulation of glucose import

From the genes related to lipid metabolic processes described in Table 7, eight were annotated with GO terms for insulin synthesis, secretion and regulation, including the *IL6* gene that exhibited a GO term for positive regulation of pancreatic β cell activation (responsible for synthesizing and secreting insulin^36^). Increases in insulin levels in chicken may affect expression of genes related to glucose and lipid metabolism^37^, consequently affecting fat accumulation. Based on these facts, we considered the genes annotated with GO terms related to insulin as PCGs for lipid metabolism and fat deposition regulation in chicken.

Several genes, namely *PLA2G1B, SELM, DOK5, HTR2A*, and *GDF3* have been previously associated with obesity^38–42^. The *PLA2G1B* gene harbored a SNP associated with fat accumulation and distribution in humans^38^. In mice, the knock-out of *SELM* gene resulted in elevated white adipose tissue deposition^39^. In the *DOK5* gene, genetic variants were associated with obesity in North Indian patients^40^. In the *HTR2A* gene, polymorphisms were associated with central adiposity in a study with humans^41^. Mice that were *GDF3* deficient exhibited a modest reduction in adiposity^42^. These studies corroborate the selection of these genes as PCGs for fat deposition.

Four genes were located within selection signature regions previously identified^21^ in founders of the F_2_ population used in this study: *CHST11*, *NR4A2*, *GPD2* and *INSR* (Supplementary Fig. S4), indicating that SNPs in these genes exhibit frequency differences between the parental lines, and may be associated with fat deposition. Thus, these genes exhibits additional evidences to support their selection as candidates, even considering that the selection signature regions did not overlap with the SNPs with the highest model frequency in each window (Fig. 2); in most cases, are located nearby. Moreover, *NR4A2*, *GPD2* and *INSR* were located within haplotype blocks that harbored the SNP with the highest model frequency within the genomic window associated, providing additional information in support of the PCGs.

The *CHST11* gene is associated with lipid metabolism, and its expression affects lipid accumulation in adipocytes^43^. The *NR4A2* gene encodes a member of the steroid-thyroid hormone-retinoid receptor superfamily. NR4A receptors regulate hepatic glucose^44^, and consequently, lipid metabolism. Additionally, this gene influences retinoid signaling^44–46^, and although the mechanisms have still not been clearly elucidated, it is known that retinoid plays an important role in lipid metabolism^47^.

The protein encoded by *GPD2* gene acts on the mitochondrial membrane, and its expression may affect gluconeogenesis and glucose homeostasis^48^. In a study with mice, Brown *et al*.^49^ reported a reduction of 40% in the weight of white adipose tissue in the individuals with knocked-out *GPD2*. *NR4A2* and *GPD2* genes overlapped with one selection signature region (Supplementary Fig. S4), indicating that selection possibly affected the frequency of SNPs in both genes. These SNPs may be associated with glucose homeostasis and lipid metabolism.

The *INSR* gene plays an important role in insulin signaling^50^, and as mentioned before, insulin levels affect lipogenesis, and consequently, lipid accumulation. Additionally, the *INSR* gene overlapped with a selection signature region previously reported (Supplementary Fig. S4) with genetic variants mainly fixed in the broiler line^21^. These findings indicate that selection could affected the frequency of SNPs in this PCG leading to changes in lipogenesis and lipid accumulation in chickens.

In humans, *CRY1* is expressed in subcutaneous abdominal and visceral fat depots^51^. In chicken, the *CRY1* gene is located 1.5 kb from a selection signature region previously identified in chicken by our group^13^ (Supplementary Fig. S4). Genetic variants located in this gene may have their frequencies affected by selection^52^.

Seven other genes (*MB*, *SLC7A1*, *SLC1A6*, *SLC25A42*, *SLC5A5*, *SLC39A3* and *SLC35E1*) were not annotated with GO terms related to fat deposition but were considered PCGs for fat deposition for the reasons explained below. Myoglobin (*MB)* expression was associated with fatty acid metabolism in a study with mice^53^. Members of the solute carrier (SLC) family encodes membrane-bound transporters^54^, and one of these has been associated with obesity in humans^55,56^. Additionally, the *SLC1A6* gene belongs to the SLC1 family that regulates glutamate transport, and in liver cells, this amino acid is a precursor of fatty acid biosynthesis^57^. In a study with rats, mice and rabbits, Collin *et al*.^58^ demonstrated that glutamate transporter activity might regulate energy balance. Energy balance is directly related with fatty acid biosynthesis and consequently, fat storage.

Some positional candidate genes were considered based on their function on lipid metabolism in mammals, because no information was available in chickens. Although many studies reported that the function of some genes can be different in chickens compared to mammals^59–65^, Li *et al*.^59^ reported differentially expressed genes consistent with conservation of lipid metabolism and adipogenesis processes in chicken and mammal. We performed an investigation for potential candidate genes based on GO terms and literature information, and we considered those genes as candidates for fat deposition regulation in chickens. We suggest further functional studies to validate our findings.

### SNPs annotated in PCGs genes

The density of sequence SNPs in our PCGs ranged from 13 to 116 SNPs/kb, with an average of 40 SNPs/kb. Previous studies in chicken reported average density of SNPs across the entire genome ranging from 5 to 78 SNPs/kb^50,66^, corroborating our findings. The top three genes with the highest SNP density were *SELM, PLA2G1B* and *SLC39A3* (Table 4). These genes should be thoroughly investigated, since this variability may be affecting fat deposition in the F_2_ Chicken Resource Population.

Genetic variants in PCGs that overlapped with selection signature regions may exhibit polymorphisms responsible for phenotypic variation^67–69^. Moreover, haplotype blocks that harbor the SNP with the highest model frequency can carry the causative mutation^70,71^. Thus, the genetic variants annotated in potentially functional gene regions from *CRY1*, *CHST11*, *NR4A2*, *GPD2* and *INSR* are important candidates for further association and functional studies.

Approximately 65% of the sequence SNPs detected in our PCGs were located in intronic regions (Fig. 3). Intronic SNPs can modulate gene expression, and consequently, affect the phenotype^72,73^. However, they are commonly deemed as potentially neutral. The other 35% of the SNPs detected were annotated in functional gene regions: up/downstream from the gene, 3’and 5’-UTRs, exons (synonymous and non-synonymous), and splice sites (Fig. 3).

Variants in coding regions can be related to phenotypic variation, and, more specifically, non-synonymous variants imply amino acid changes^74^. Changes in amino acids can potentially affect protein function. To predict whether SNPs in coding regions are deleterious or not (may affect the protein function), we predicted the SIFT score as described in the Methods section.

Twelve potentially deleterious SNPs were identified in eight PCGs (Table 6), and most of them are located in PCGs involved in lipogenesis, levels of triglycerides and obesity. Moreover, two of these genes exhibited more than one deleterious mutation: *NR4A2*, involved in the regulation of hepatic glucose affecting lipid metabolism, and *TM6SF2*, involved in the regulation of triglyceride levels in the liver. Changes in the function of these genes may affect fat deposition in chicken. All deleterious mutations are important candidates for further association and functional studies.

From the 12 potentially deleterious SNPs, only rs315426765 SNP on the *TM6SF2* gene was present in the Affymetrix genotyping array. However, that SNP was removed after genotyping quality control due to low MAF. The search for potential candidate SNPs identified previously from whole genome sequence data in similar chicken populations allowed us to identify a great number of SNPs, which are not present in the Affymetrix genotyping array, improving our chances to identify potentially causative mutations.

In summary, our study identified 22 unique QTLs for abdominal fat and carcass fat content, and approximately 40% of the QTLs detected were considered novel QTLs for the traits analyzed. The 22 QTLs detected harbored 26 PCGs that were involved in biological processes of fat deposition. Three of these 26 PCGs were located within haplotype blocks that were associated with fat traits and five of these 26 genes overlapped with selection signature regions. From the total number of SNPs annotated in PCGs, approximately 35% were in functional regions, and from those 12 were predicted to be deleterious variants. The *NR4A2* gene is a strong candidate for fat deposition regulation in chicken, since it is within a QTL for carcass fat content traits, is in linkage disequilibrium with the SNP with the highest model frequency, is under selection in the founder lines, and contains three potential causal SNPs.

GWAS using a high density of SNPs allowed us to map QTLs with better resolution than previously done with microsatellite markers, thus facilitating the search for PCGs. The integration of haplotype blocks detection, selection signature regions and potentially deleterious SNPs allowed us to refine the list of PCGs for fat deposition traits. The PCGs identified, especially those within the haplotype blocks, overlapped with selection signature regions and harboring genetic variants located on potentially functional gene regions, are strong candidates for selection in poultry breeding programs aiming to improve the accuracy of selection and reduce excessive fat deposition. Further functional validation studies could be helpful to understand the role of the candidate genes and genetic variants associated with fat deposition regulation.

## Methods

All experimental protocols related to animal experimentation in this study were performed in agreement with resolution number 010/2012 approved by Embrapa Swine and Poultry Ethics Committee on Animal Utilization to ensure compliance with international guidelines for animal welfare.

### Chicken Population

A total of 529 chickens from the Embrapa F_2_ Chicken Resource Population were genotyped (28 parental chickens from layer and broiler lines; 5 chickens from F_1_ and 496 chickens from the F_2_-TCTC generations). This F_2_ population is the result of crosses between two closed parental lines: a broiler line (called TT) and a layer line (called CC). The TT line had been selected for higher body weight, feed conversion, carcass and breast yield, viability, fertility, hatchability, reduction of abdominal fat weight and metabolic syndromes. The CC line had been selected for egg production, egg weight, feed conversion, viability, sexual maturity, fertility, hatchability, egg quality and low body weight. More details about the Embrapa F_2_ Chicken Resource Population were described by Rosário *et al*.^30^. All the birds from the Embrapa F_2_ Chicken Resource Population were reared as broilers with free access to water and a corn and soybean meal-based diet^33^. More details of the diet content can be found on Nones *et al*.^9^.

This F_2_-TCTC generation was previously used to map numerous QTLs for performance, carcass and chemical component traits^8–10,31,33,75^ using microsatellite markers. For the study reported in this paper, selection of families for genotyping and GWAS was based on F1 males that appeared to be heterozygous for QTL effects reported in previous studies.

### Phenotype measurement

A total of 502 chickens from the F_2_ population were slaughtered and eviscerated at 42 days of age, after 6 h of fasting to avoid contamination of the carcass during slaughter and post-slaughter processing. The body weight at 42 days of age (BW42) was measured and carcasses were stored at 4 °C for 6 h. Then, the carcass and its cuts were weighed. Abdominal fat was removed from chilled carcass for weighing (abdominal fat weight, ABF). Abdominal fat percentage (ABFP) was calculated dividing ABF by BW42 and multiplying by 100^10^.

Fat (ether extract) was measured by near-infrared reflectance spectroscopy (NIRS) and estimated as percentage according to the weight of each sample (250 g of ground and homogenized carcass)^9^. Carcass fat content weight (CFC) was estimated by multiplying the percentage in the sample by BW42. Carcass fat content on dry matter basis (CFCDM) was estimated dividing sample fat by carcass dry matter content and multiplying by 100. More details about trait measurement can be found in Campos *et al*.^10^, Nunes *et al*.^3^ and Nones *et al*.^9^.

### DNA extraction, genotyping and quality control

Genomic DNA was extracted from blood samples with DNAzol® protocol. After extraction, DNA integrity was evaluated on agarose gel (1%), quantified in spectrophotometer NanoDrop^®^ (Thermo Fisher Scientific), and diluted to the final concentration of 20 ng.µL^−1^. Diluted genomic DNA was prepared for genotyping following Affymetrix protocols and genotyped with the 600 K Axiom Chicken Genotyping Array. That array comprises SNPs chosen to be segregating for different chicken lines^66^.

Quality control analysis and genotype calling were performed with Affymetrix Power Tools v1.17.0 (APT). Samples that exhibited DishQC ≥0.82 and call rate ≥90% were kept for further analysis. Among those high-quality samples, the most accurate and polymorphic marker SNPs, with call rate ≥98% and minor allele frequency (MAF) ≥2%, were kept for further analysis. In this step, R scripts from the package SNPolisher were used. SNPs located in the sex chromosomes, unmapped linkage groups, without genomic annotation and those that were monomorphic were removed from the analysis. SNP annotation was based on the Gallus_gallus-5.0 chicken assembly (*NCBI*). Missing genotypes were replaced by their average covariate value at that locus^76^.

### Descriptive statistics and heritability

The mean and standard deviation of each phenotype were calculated using in-house scripts in R software (http://www.r-project.org/). The estimation of variance components was performed using a Bayes C model in GenSel software^18^. The resultant posterior means of the variance components were used as *priors* in a Bayes B model to estimate genomic heritability for each trait using GenSel. Sex and hatch were included as fixed effects in the model, and BW42 was used as a fixed covariate for ABF and CFC.

### Genome-wide association analysis

The SNPs retained after quality control were used in the GWAS analysis with a Bayesian approach, performed with the GenSel software^18^. In the first step, a Bayes C model was used to estimate the genetic and residual variances, and these values were used to run a Bayes B model, as performed by Cesar *et al*.^76^. The Bayes B model samples the effects of SNPs assuming that some fraction of their effects are zero and with unequal variance of each effect^77^. The mathematical model presented below was used in the association analysis:

$${\boldsymbol{y}}={\boldsymbol{Xb}}+\,\sum _{j=1}^{k}{{\boldsymbol{a}}}_{j}{\beta }_{j}{\delta }_{j}+{\boldsymbol{e}},$$

In this model, ***y*** represents the vector of phenotypic values; ***X*** is the incidence matrix for fixed effects; ***b*** is the vector of fixed effects; *k* is the number of SNPs; **a**_***j***_ is the column vector representing the SNP as a covariate in locus _j_ coded with the number of B alleles; *βj* is the random substitution effect for locus *j* assumed to be normally distributed *N* (0, *σ*^2^_*βi*_) when *δj* = 1 but *βj* = 0 when *δj* = 0, with *δj* being a random variable 0/1 indicating the absence (with probability π) or presence (with probability 1-π) of the locus *j* in the model, and ***e*** is the vector of residual effects assumed to be normally distributed *N* (0, *σ*^2^_*e*_). Sex and hatch were included as fixed effects in the model, and BW42 was used as a fixed covariate for ABF and CFC.

We assumed π = 0.9988 in the Bayes B models and obtained 41,000 Markov Chain Monte Carlo (MCMC) samples with the first 1,000 samples being discarded. A map file was used to position the SNPs into 943 non-overlapping windows of 1 Mb. We adopted 1 Mb non-overlapping windows in our study in agreement with other recent GWAS studies in chickens reported in the literature using genomic prediction methodology^19,20,78,79^. Many SNPs were fitted simultaneously in the model and due to high linkage disequilibrium, the QTL effect can be distributed across these markers^18^, and these previous studies showed that the 1 Mb windows can capture the effects.

Each window is expected to explain 0.106% of the genetic variance (100%/943) based on an infinitesimal model^20^, and windows that explained five times more than the expected value (0.53%) were considered to be biologically significant. Thus, we selected only biologically significant windows to characterize and identify PCGs. Additionally, within each significant window (QTLs), we selected the SNP most frequently included in the model.

### Overlap with previously mapped QTLs

We checked the overlaps of all genomic windows detected with QTLs previously mapped in chicken^7^, using Chicken QTLdb - release 33, accessed in September, 2017. We used the search tool in Chicken QTLdb website, using our QTL coordinates based on Gallus_gallus-5.0 chicken genome assembly. Previously mapped QTLs overlapped were reported by their respective QTL ID numbers. The genomic windows that did not overlap with previously annotated QTL regions were considered novel discoveries.

### Identification of candidate genes, overlap with selection signature regions, haplotype blocks and SNP screening

The goal of this study was to investigate PCGs based on literature information to provide new insights for further studies, and also to better understand the genetic architecture of fat deposition traits. In this context, the list of annotated genes within each associated genomic window were searched using NCBI and OMIM databases, the BioMart tool and literature to find GO terms and biological processes related to abdominal fat, lipid metabolism, fat content, and fat deposition. For all the analyses, we considered the gene annotation from *Ensembl Genes 90 Database* and the Gallus_gallus-5.0 (NCBI) chicken genome assembly.

To refine the list of candidate genes, we compared our list of PCGs against selection signature regions identified in a previous study with 28 parental chickens from the two lines that generated the F_2_ population analyzed in our study^21^. That study used whole genome sequence to identify genetic variants and applied the Fst method^21,80^ to estimate the divergence between populations and identify regions under selection (TT *vs*. CC lines). We used the CrossMap tool (http://crossmap.sourceforge.net/) to convert selection signature coordinates (from Gallus_gallus-4.0) to the Gallus_gallus-5.0 chicken genome assembly (NCBI).

We also compared our list of PCGs against the haplotype blocks that harbored the SNPs with the highest model frequency within each associated genomic window. PLINK v.1.9^81^ software was used to detect the haplotype blocks, with default parameters.

Additionally, to identify potential candidate genetic variants for fat deposition in chicken, we performed a screening of SNPs located in PCGs, using the same dataset of sequencing SNPs used to detect the selection signature regions (all the 13 million SNPs from NGS data identified were submitted to dbSNP-NCBI with the handle “LBA_ESALQ”)^21^.

In order to refine our list of PCG variants, we searched for genetic variants predicted as deleterious and high impact. To predict whether SNPs in coding regions are deleterious or not (may affect the protein function), we obtained the SIFT (sorting intolerant from tolerant) scores. That score is an assessment of the level of conservation in homologous protein sequences^82^ implemented by the VEP tool^83^. SIFT scores were predicted for all non-synonymous and stop codon (gained/lost) variants located in the PCGs.

The prediction of high impact SNPs was also performed using the VEP tool^83^ that provides an estimation of the putative impact of the variant classified as high impact, i.e. annotating all the mutations annotated as transcript ablation, splice acceptor, splice donor, stop gained, frameshift, stop loss, start lost and transcript amplification, mutations that may cause protein truncation, loss of function or trigger nonsense mediated decay (http://www.ensembl.org/info/genome/variation/predicted_data.html).

## Electronic supplementary material

Supplementary Information File

Supplementary Spreadsheet S1

Supplementary Spreadsheet S2

Supplementary Spreadsheet S3

## Data Availability

All SNPs reported (identified by sequencing) were submitted to dbSNP-NCBI with the handle “LBA_ESALQ”. The datasets used and/or analysed during the current study (genotypes and phenotypes) are available from the corresponding author on reasonable request.
