# Supplementary material for: Integration of genome wide association studies and whole genome sequencing provides novel insights into fat deposition in chicken
Source: Sci Rep. 2018 Nov 1;8:16222. doi: 10.1038/s41598-018-34364-0 (PMC6212401; doi:10.1038/s41598-018-34364-0)
Supplement: Supplementary file 1 — Supplementary Information File [file 41598_2018_34364_MOESM1_ESM.docx]

**Integration of genome wide association studies and whole genome sequencing provides novel insights into fat deposition in chicken**

Gabriel Costa Monteiro Moreira^1^; Clarissa Boschiero^1^; Aline Silva Mello Cesar^1^; James M Reecy^2^; Thaís Fernanda Godoy^1^; Fábio Pértille^1^; Mônica Corrêa Ledur^3^; Ana Silvia Alves Meira Tavares Moura^4^; Dorian J Garrick^5^ & Luiz Lehmann Coutinho^1*^

^1^ Department of Animal Science, University of São Paulo, Piracicaba, SP, 13418-900,

Brazil

^2^ Department of Animal Science, Iowa State University, Ames, IA, 50011, USA

^3^ Embrapa Suínos e Aves, Concórdia, SC, 89715-899, Brazil

^4^ FMVZ / São Paulo State University, Botucatu, SP, Brazil.

^5^ School of Agriculture, Massey University, Ruakura, Hamilton, New Zealand

*Corresponding author:

Email: llcoutinho@usp.br (LLC)

**SUPPLEMENTARY INFORMATION**

**Supplementary figures**

Supplementary Fig. S1. Manhattan plot of the posterior means of the percentage of genetic variance explained by each 1 Mb SNP window across the 28 autosomal chromosomes for ABF (abdominal fat in grams): (A) genomic windows located on macrochromosomes, and (B) genomic windows located on microchromosomes.

Supplementary Fig. S2. Manhattan plot of the posterior means of the percentage of genetic variance explained by each 1 Mb SNP window across the 28 autosomal chromosomes for CFCDM (carcass fat content on dry matter basis): (A) genomic windows located on macrochromosomes, and (B) genomic windows located on microchromosomes.

Supplementary Fig. S3. Manhattan plot of the posterior means of the percentage of genetic variance explained by each 1 Mb SNP window across the 28 autosomal chromosomes for ABFP (abdominal fat percentage): (A) genomic windows located on macrochromosomes, and (B) genomic windows located on microchromosomes.

Supplementary Fig. S4. Plot showing the overlapping of selection signature regions and positional candidate genes (PCGs). Fst regions means selection signature regions. (a) Plot of the region around CRY1 gene (GGA1, 53,603,247-53,803,224 bp) showing the selection signature region nearby CRY1 gene. (b) Plot of the region around CHST11 gene (GGA1, 53,603,247-53,803,224 bp) showing the overlap of two selection signature regions. (c) Plot of the region around NR4A2 and GPD2 genes (GGA7, 36,195,323-36,295,960 bp) showing the overlap of one selection signature region. (d) Plot of the region around INSR gene (GGA28, 4,051,975-4,211,970 bp) showing the overlap of one selection signature region. The genome positions of selection signatures regions are: GGA1 from 53,678,235 to 53,728,228, from 54,470,039 to 54,510,038, and from 54,570,039 to 54,590,038; on GGA7 from 36,220,480 to 36,270,797; on GGA28 from 4,111,972 to 4,151,970.

**Supplementary Spreadsheets**

Supplementary Spreadsheet S1. Characterization of the SNPs with higher model frequency within each genomic window associated.

Supplementary Spreadsheet S2. Haplotype blocks detected harboring SNPs with highest model frequency in each genomic window associated.

Supplementary Spreadsheet S3. Characterization of all 943 1-Mb genomic windows including their respective proportion of genetic variance explained and their posterior probability association (PPA). Each tab represents the respective analyzed trait.

**Supplementary figures**

Supplementary Fig. S1.

Supplementary Fig. S2.

Supplementary Fig. S3.

Supplementary Fig. S4.
